# Chemotherapy treatments, costs of care, and survival for patients diagnosed with small cell lung cancer: A SEER‐Medicare study

**DOI:** 10.1002/cam4.2626

**Published:** 2019-10-31

**Authors:** Changxia Shao, Jinghua He, Sumesh Kachroo, Fan Jin

**Affiliations:** ^1^ Merck & Co., Inc Kenilworth NJ USA

**Keywords:** drug therapy, resources/economics/utilization, SEER program, treatment outcomes

## Abstract

**Objectives:**

The effectiveness and costs of new treatments should be assessed in relation to existing practice. We describe treatments, survival and costs for advanced or metastatic small cell lung cancer (SCLC) patients receiving systemic therapy in the period preceding the introduction of immunotherapies.

**Materials and Methods:**

This was a retrospective cohort study of patients aged ≥65 years, identified using linked Surveillance, Epidemiology, and End Results and Medicare databases. Individuals with a new primary diagnosis of SCLC between January 2007 and December 2013 were followed until December 2014. Chemotherapy treatments, health care visits and costs (in 2016 USD), and survival were determined by line of therapy.

**Results:**

A total of 11 812 patients were identified with SCLC. First‐line (1L) chemotherapy was received by 6509 (55.1%) patients, most (93.2%) with carboplatin‐ (71.0%) or cisplatin‐ (22.2%) based therapies, typically combined with etoposide (79.2%). Second‐ (2L) and third‐ (3L) line chemotherapies were received by 2238 (18.9%) and 679 (5.7%) patients, of which 48.4% and 30.9%, respectively, were platinum‐based. The median durations of 1L, 2L, and 3L carboplatin‐based therapies were 5.9, 4.8, and 5.4 months, respectively, and the corresponding durations of cisplatin‐based therapies were 5.3, 4.2, and 5.3 months. During 1L, 2L, and 3L chemotherapies, patients averaged 8.2, 7.4, and 7.3 health care visits per month, respectively, and incurred total mean health care costs of $60 223, $42 636, and $35 903 per patient, respectively. Median survival from the start of 1L, 2L, and 3L chemotherapy was 9.2, 6.0, and 5.7 months, respectively.

**Conclusion:**

First‐line chemotherapy was primarily platinum‐based, and a plethora of different regimens was used for 2L and 3L chemotherapies. Median survival from the start of 1L chemotherapy was 9 months, with an associated health care cost of $60 000. These data highlight an unmet medical need among SCLC patients receiving systemic therapy.

## Graphical abstract

The objective of this manuscript was to describe the existing practice of small cell lung cancer (SCLC) care as a real‐world benchmark with which to assess the performance of the anticipated new immunotherapies. First‐line chemotherapy was largely platinum‐based, consistent with US guidelines while a variety of regimens were used for second‐ and third‐line chemotherapies, reflecting the lack of an accepted standard of care for these patients. Median survival from the start of each line of chemotherapy was <12 months. The data presented in this manuscript provide a historical benchmark with which to assess the costs of the anticipated new immunotherapies and their effects on survival, as well as on the burden of SCLC on patients and on the health care system.

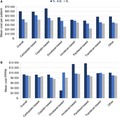

## 1 INTRODUCTION

Small cell lung cancer (SCLC) is a distinct subtype of lung cancer, comprising 13.1% of all lung cancer histologic types, and representing 29 650 cases annually.^1^ Relative to non‐small cell lung cancer, SCLC is characterized by a rapid growth and early, wide‐spread metastases, often involving the liver, adrenal glands, bones, and/or brain.^2^, ^3^ Almost all cases of SCLC are attributable to cigarette smoking.^4^

Standard chemotherapies for patients with SCLC have not changed significantly during the last three decades until recent immunotherapies.^5^ The National Comprehensive Cancer Network (NCCN) guidelines recommended cisplatin or carboplatin in combination with etoposide as a primary or adjuvant systemic therapy for patients with limited stage disease (corresponding to the American Joint Committee on Cancer's [AJCC] stages I‐III).^6^ Treatment of extensive‐stage (corresponding to AJCC stage IV) SCLC is palliative.^7^ Historically, first‐line (1L) therapy for extensive‐stage SCLC in the United States was a combination of cisplatin or carboplatin with etoposide or irinotecan.^6^

Although SCLC is responsive to initial treatment, most patients relapse with relatively resistant disease.^8^ After relapse, patients have a median survival of 4‐5 months when treated with further chemotherapy, with the likelihood of response highly dependent on the time from initial therapy to relapse.^9^ If the time between initial therapy and relapse is less than 3 months, response to most agents or regimens is poor (≤10%). If the time interval is more than 3 months, expected response rates are approximately 25%.^10^ Subsequent treatment (when relapse is 6 or fewer months after initial therapy) is with a variety of chemotherapies, including but not limited to topotecan, irinotecan, paclitaxel, or docetaxel.^6^ When relapse is more than 6 months after initial therapy, treatment with the original regimen is recommended.^6^

Recently, a number of immunotherapies—specifically, different types of programmed cell death inhibitors—have received approval by the US Food & Drug Administration (FDA) for different indications across a wide range of tumor types.^11^ The recent successes of immunotherapy in the treatment of other cancer types suggest that immunotherapies may provide a clinical benefit that has not yet been achieved for SCLC patients by traditional chemotherapy treatments.^8^ In 2018 nivolumab was approved for metastatic SCLC after disease progression following platinum‐based therapy and at least one other line of therapy; and in 2019 atezolizumab plus carboplatin and etoposide was approved for 1L therapy among patients with extensive‐stage SCLC.^12^, ^13^ Several other immunotherapies are in advanced stages of clinical development for SCLC, including pembrolizumab (approved for 3L SCLC and recommended in the 2019 NCCN guidelines for relapse ≤6 months^10^), ipilimumab, tremelimumab, and durvalumab.^14^ The performance of these new treatments should be assessed in relation to the existing standard chemotherapies.^8^, ^15^

The objective here is to describe the existing practice of SCLC care as a real‐world benchmark with which to assess the performance of the anticipated new immunotherapies.

## 2 MATERIALS AND METHODS

### 2.1 Study design

This was a retrospective cohort study of patients identified in the linked Surveillance, Epidemiology, and End Results (SEER) and Medicare databases with a new primary diagnosis of SCLC. Chemotherapy treatments, health care visits, total costs, and survival were determined. As this was a retrospective study of a deidentified data set, individual patient informed consent was not required.

**Figure 1:**
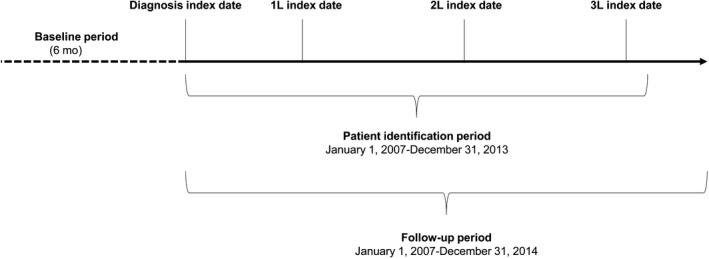
Diagram of study index dates and time periods

Figure 1 shows the study time periods and index dates. Patients were identified on a diagnosis index date occurring during a patient identification period, from 1 January 2007 to 31 December 2013, and tracked within a follow‐up period, to 31 December 2014. The diagnosis index date was the date of primary diagnosis of SCLC in the SEER database. A baseline period was defined as the 6‐month period prior to the index diagnosis date. The first‐line (1L), second‐line (2L), and third‐line (3L) index dates were the dates of initiation of the respective chemotherapy treatment lines. The patient follow‐up period began on the diagnosis index date and continued for overall survival outcomes until the earliest event of death, last SEER contact date, or 31 December 2014. Survival, duration of treatment, and time to next treatment were estimated using the Kaplan‐Meier method. For other outcomes (health care use and costs), follow‐up continued until the earliest event of death, disenrollment from Medicare Fee‐for‐Service (FFS), or 31 December 2014.

### 2.2 SEER‐Medicare data set

The linked SEER‐Medicare database combines clinical information from population‐based cancer registries with insurance claims information from the Medicare program.^16^ It contains files for patients who reside in one of the geographic areas covered by the SEER registries, who are ≥65 years of age (ie, who are Medicare eligible), and who have been diagnosed with cancer. The SEER and Medicare data sets are linked via an algorithm that matches social security number, name, sex, and date of birth. Patient identifiers (ie, name and social security number) are replaced by an anonymous record number, which allows linkage of patients over time. As of 2017, the SEER‐Medicare linked database included incident cancer cases diagnosed between 1991 and 2013, their Part A and Part B Medicare claims between 1991 and 2014, and Part D pharmacy claims between 2007 and 2014. In this study, cancer characteristics and patient survival (time and cause of death) were determined using the SEER Patient Entitlement and Diagnosis Summary File. Patient demographic information, comorbidities, treatment patterns, health care use, and costs of care were determined from Medicare claims data.

### 2.3 Study sample

Patients were included in the study if they met the following criteria: they received a diagnosis of SCLC as first primary diagnosis between 1 January 2007 and 31 December 2013; were ≥65 years of age on the diagnosis index date, and were continuously enrolled in Medicare Part A and B Medicare FFS coverage during the 6‐month baseline period and the month of the index diagnosis date. Patients were excluded if: multiple primary cancers were recorded in the SEER cancer registry during the patient identification period; the cancer diagnosis was reported exclusively by death certificate or autopsy; the patient was 64 years of age or younger on the diagnosis index date; the month or year of the index diagnosis date were not recorded; the stage at diagnosis was not recorded; the available baseline period was less than 6 months.

### 2.4 Measurements

Small cell lung cancer was identified by SEER codes C34.0‐C34.9, histology from codes 8041/3, 8042/3, 8043/3, 8044/3, 8045/3. Survival was calculated from the index date (diagnosis, 1L, 2L, or 3L) to the date of death, the end of follow‐up, or 31 December 2014, whichever came first. Comorbidities were assessed during the 6‐month baseline period using a standard SEER‐Medicare program based on the Charlson comorbidity index. The program identifies 18 comorbid conditions (other than cancer) by International Classification of Disease‐ 9th Edition code in the Medicare inpatient and outpatient claims data, assigns a prespecified weight to each comorbid condition, and computes an overall score.

Chemotherapies were identified within a time window, from 30 days prior to the diagnosis index date, up to the end of the follow‐up period. They were identified by National Drug Code and/or Healthcare Common Procedure Coding System codes from a comprehensive list of chemotherapy drugs. Chemotherapy agents received within 90 days of the surgery date were considered neoadjuvant/adjuvant therapy and were excluded from the definition of 1L treatment. A combination regimen was designated by the addition of one or more chemotherapies within 28 days of the line of therapy index date. Dropping a drug from a combination regimen did not advance the line of treatment. The duration of chemotherapy treatment was the time interval between the 1L, 2L, or 3L index date and the date on which the patient either switched to another treatment regimen or to surgery, experienced a 90‐day treatment gap, or the end of follow‐up. The time to next treatment was the time interval between initiation of two adjacent treatment lines or from the last treatment line to the end of follow‐up.

Health care utilization and medical costs associated with chemotherapy were assessed for each line of therapy from the therapy index date until treatment end. The following were included in the measurements: hospitalizations, emergency room and outpatient visits, and use of nursing facilities, home health care, hospices, and durable medical equipment. Costs were the sum of Medicare costs (Medicare payments to the service provider), other payer costs (coinsurance reimbursements), and patient liability costs (deductibles and copays), and were expressed as total costs and costs per patient per month (PPPM).

### 2.5 Data analysis

The study was descriptive in nature and no hypotheses were tested. All outcomes were stratified by line of therapy (1L, 2L, 3L). Specific chemotherapy regimens were reported as patient number and percent. The months' duration of each chemotherapy regimen and time to next treatment were reported as median (95% confidence interval [CI]). Health care use and costs associated with each line of chemotherapy were calculated per patient during each line of treatment. Multiple claims of the same type on the same day were considered as a single visit. The number of visits per patient and number of visits PPPM were reported as mean (SD). All costs were adjusted to 2016 US dollar (USD) using the Consumer Price Index. Survival from the index therapy date (1L, 2L, and 3L) was determined using the Kaplan‐Meier product‐limit method and stratified by the most commonly used regimens. Survival was defined as the time from the therapy index date (1L, 2L, and 3L) to the date of death due to any cause. Patients without observed death were censored at the earlier date of either last SEER contact or 31 December 2014. Median survival (in months) and associated 95% CIs were reported.

## 3 RESULTS

### 3.1 Patients

A total of 11 812 patients were included in the study. The mean age at diagnosis was 74.6 years, 51.9% were female, 66.0% were diagnosed at stage IV and 27.6% at stage III (Table 1). The characteristics of patients who did and did not receive 1L chemotherapy were similar (Table 1). The characteristics of the 7797 patients who were diagnosed with stage IV cancer were similar to those of the overall population (Table S1). The median follow‐up time was 11.2 months for those who received 1L chemotherapy and 2.0 months for those who did not (Table 1).

**Table 1:** Patient demographic and clinical characteristics^a^

	Overall (N = 11 812)	No 1L chemotherapy (N = 5303)	1L (N = 6509)	2L (N = 2238)	3L (N = 679)
Age, mean y (SD)	74.6 (6.2)	75.9 (6.6)	73.5 (5.7)	72.7 (5.3)	72.1 (5.1)
Female	6131 (51.9)	2744 (51.7)	3387 (52.0)	1143 (51.1)	346 (51.0)
Race					
White	10 592 (89.7)	4684 (88.3)	5908 (90.8)	2009 (89.8)	606 (89.2)
Black	828 (7.0)	438 (8.3)	390 (6.0)	145 (6.5)	47 (6.9)
Asian	340 (2.9)	157 (3.0)	183 (2.8)	73 (3.3)	*
Other/unknown	52 (0.4)	24 (0.5)	28 (0.4)	11 (0.5)	*
CCI score, mean (SD)^b^	0.95 (1.35)	1.08 (1.50)	0.84 (1.21)	0.76 (1.10)	0.72 (1.09)
Region					
Midwest	1710 (14.5)	745 (14.0)	965 (14.8)	296 (13.2)	100 (14.7)
Northeast	2050 (17.4)	901 (17.0)	1149 (17.7)	438 (19.6)	141 (20.8)
South	4222 (35.7)	1837 (34.6)	2385 (36.6)	777 (34.7)	214 (31.5)
West	3830 (32.4)	1820 (34.3)	2010 (30.9)	727 (32.5)	224 (33.0)
Tumor size, mean size cm (SD)^c^	5.37 (6.72)	5.55 (6.43)	5.25 (6.92)	5.19 (5.74)	5.90 (8.34)
Staging at diagnosis					
I‐II	751 (6.4)	251 ( 4.7)	500 ( 7.7)	122 ( 5.5)	32 ( 4.7)
III	3264 (27.6)	1241 (23.4)	2023 (31.1)	687 (30.7)	228 (33.6)
IV	7797 (66.0)	3811 (71.9)	3986 (61.2)	1429 (63.9)	419 (61.7)
Follow‐up, mo					
Median	6.1	2.0	11.2	15.2	19.3
Mean (SD)	10.2 (12.4)	4.5 (7.7)	14.8 (13.5)	18.2 (12.1)	22.5 (11.9)

Abbreviations: 1L, first line; 2L, second line; 3L, third line; CCI, Charlson Comorbidity Index; SD, standard deviation.

a Values are presented as N (%) unless otherwise indicated.

b Mean CCI score presented in 2014 macro.

c Data available for 7988 (67.6%) patients, 3286 (62.0%) patients receiving no chemotherapy, 4702 (72.2%) patients receiving 1L therapy, 1623 (72.5%) patients receiving 2L therapy, and 512 (75.4%) patients receiving 3L therapy.

* Cell counts are suppressed according to CMS cell size suppression policy.

Over half (55.1%) of the 11 812 patients received 1L chemotherapy, 2238 (18.9%) went on to receive 2L, and 679 (5.7%) subsequently received 3L chemotherapy (Table 1). Similarly, among patients diagnosed with stage IV cancer, 51.1% received 1L chemotherapy, 18.3% went on to receive 2L chemotherapy, and 5.4% subsequently received 3L chemotherapy (Table S1). The characteristics of patients receiving 1L, 2L, and 3L chemotherapy were similar.

### 3.2 Chemotherapy treatments

An overwhelming majority (93.2%) of 1L chemotherapies was carboplatin‐ or cisplatin‐based (71.0% and 22.2%, respectively), primarily in combination with etoposide (Table 2). Fewer than half (48.3%) of 2L chemotherapies were platinum‐based (carboplatin‐based, 37.7%, cisplatin‐based, 10.6%), while 22.4% were based on topotecan and 11.8% on paclitaxel. The trend away from platinum‐based chemotherapies continued for 3L treatments, of which 30.9% were platinum‐based (carboplatin‐based, 21.8%, cisplatin‐based, 9.1%), while 23.3% contained paclitaxel and 18.7% topotecan.

**Table 2:** Chemotherapy treatments, by line of therapy^a^

	1L		2L		3L	
	All (N = 6509)	Stage IV (N = 3986)	All (N = 2238)	Stage IV (N = 1429)	All (N = 679)	Stage IV (N = 419)
Carboplatin‐based	4622 (71.0)	3029 (76.0)	844 (37.7)	470 (32.9)	148 (21.8)	90 (21.5)
Carboplatin monotherapy	487 (7.5)	292 (7.3)	43 (1.9)	25 (1.7)	*	*
Carboplatin + etoposide	3876 (59.5)	2562 (64.3)	535 (23.9)	282 (19.7)	56 (8.2)	30 (7.2)
Carboplatin + others	259 (4.0)	175 (4.4)	266 (11.9)	163 (11.4)	*	*
Cisplatin‐based	1445 (22.2)	699 (17.5)	238 (10.6)	128 (9.0)	62 (9.1)	41 (9.8)
Cisplatin monotherapy	90 (1.4)	44 (1.1)	12 (0.5)	*	*	*
Cisplatin + etoposide	1279 (19.6)	594 (14.9)	113 (5.0)	50 (3.5)	12 (1.8)	*
Cisplatin + others	76 (1.2)	61 (1.5)	113 (5.0)	*	46 (6.8)	*
Docetaxel‐based	*	*	39 (1.7)	26 (1.8)	27 (4.0)	17 (4.1)
Irinotecan‐based	36 (0.6)	25 (0.6)	137 (6.1)	97 (6.8)	52 (7.7)	28 (6.7)
Paclitaxel‐based	19 (0.3)	12 (0.3)	264 (11.8)	207 (14.5)	158 (23.3)	104 (24.8)
Topotecan‐based	22 (0.3)	11 (0.3)	502 (22.4)	365 (25.5)	127 (18.7)	73 (17.4)
Other	*	*	214 (9.6)	136 (9.5)	105 (15.5)	66 (15.8)

Abbreviations: 1L, first line; 2L, second line; 3L, third line.

a Values are presented as N (%).

* Cell counts are suppressed according to CMS cell size suppression policy.

First‐line chemotherapy regimens for patients diagnosed with stage IV cancer were also primarily carboplatin‐ or cisplatin‐based (76.0% and 17.5%, respectively; Table 2). Second‐line and 3L treatment trends for stage IV patients were similar to those of the overall population. In addition, treatment patterns were similar for extensive stage disease vs recurrent limited stage disease (data not shown).

### 3.3 Duration of chemotherapy

The median durations of any line of carboplatin‐ and cisplatin‐based chemotherapies were in the range 4.8‐5.9 and 4.2‐5.3 months, respectively (Table S2). The median durations of any line of paclitaxel‐ and topotecan‐based chemotherapies were in the range 4.4‐5.4 and 4.2‐5.6 months, respectively.

The median time to the next treatment or end of follow‐up for 1L, 2L, and 3L carboplatin‐based chemotherapies was 14.5, 15.7, and 14.4 months, respectively, and for 1L, 2L, and 3L cisplatin‐based chemotherapies, 18.1, 11.7, and 7.5 months, respectively (Table S3). For any line of paclitaxel‐ and topotecan‐based chemotherapies, the median time to next treatment was in the range of 7.0‐15.9 months.

### 3.4 Health care use

**Figure 2:**
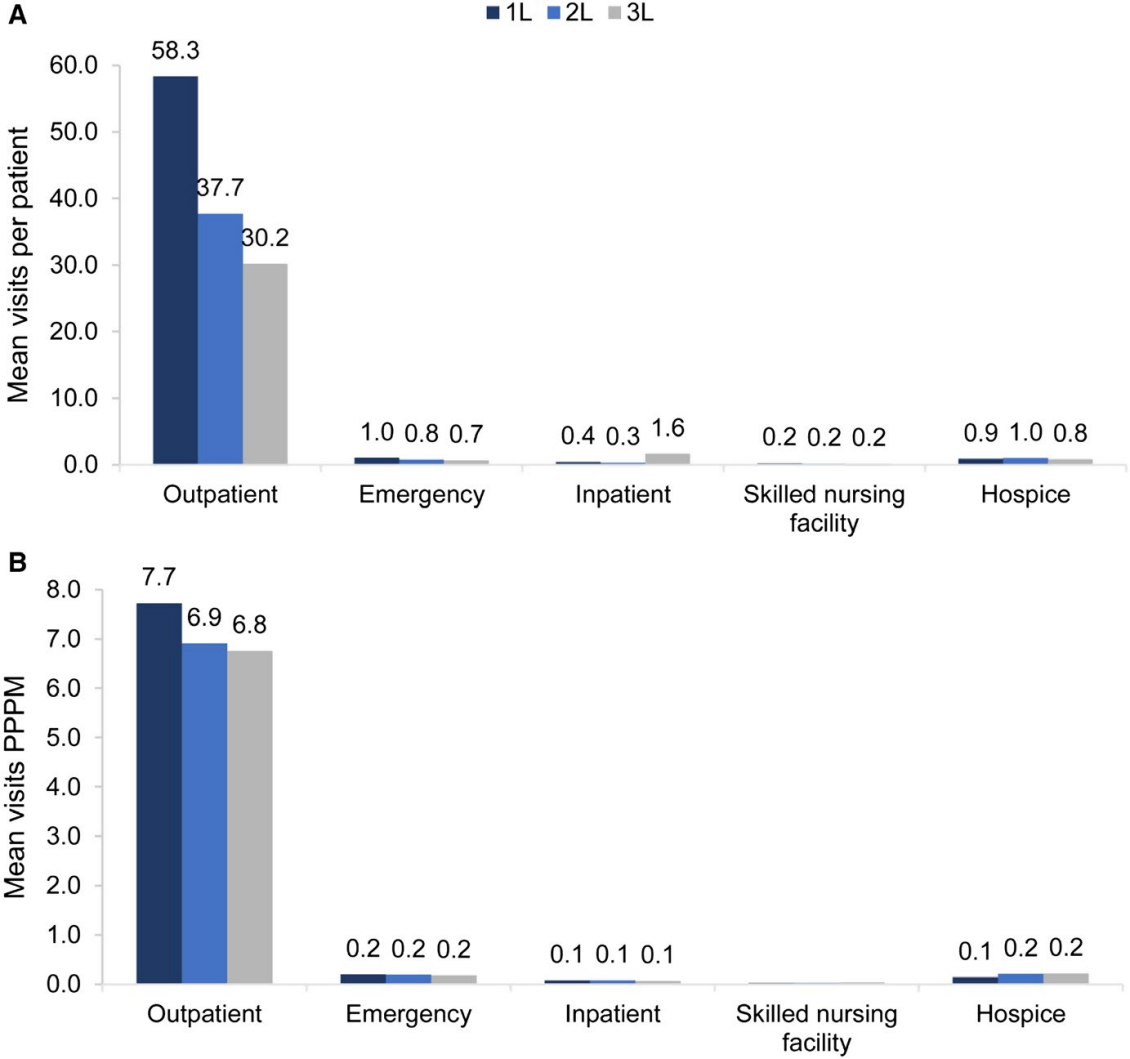
Health care use, by (A) mean visits per patients and (B) mean visits PPPM, by line of therapy. 1L, first line; 2L, second line; 3L, third line; PPPM, per patient per month

Health care visits were overwhelmingly for outpatient care, accounting for 95.8%, 94.4%, 94.1% of visits during 1L, 2L, and 3L chemotherapy, respectively. Patients receiving 1L, 2L, and 3L therapy visited the outpatient clinic 7.7, 6.9, and 6.8 times per month, respectively, for totals of 58.3, 37.7, and 30.2 outpatient visits (Figure 2).

### 3.5 Health care costs

**Figure 3:**
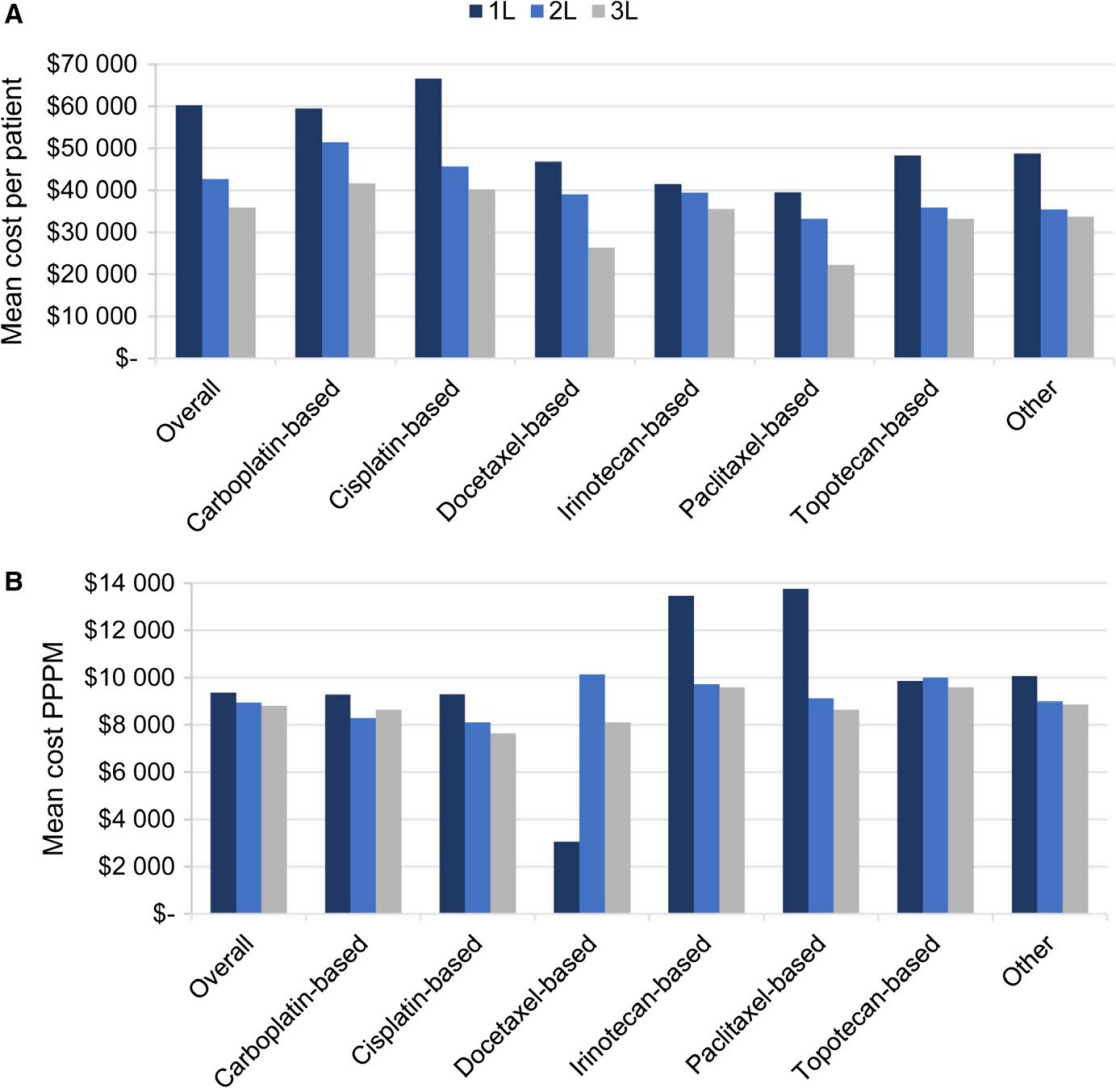
Mean cost per patient, by line of therapy and therapy type (A) overall and (B) per patient, per month. Costs presented in 2016 USD. 1L, first line; 2L, second line; 3L, third line; PPPM, per patient per month

Mean costs of health care PPPM for 1L, 2L, and 3L chemotherapy were $9373, $8942, and $8804, respectively, for total mean costs per patient of $60 223, $42 636, and $35 903 (Figure 3). Outpatient visits were the largest component of total costs, followed by emergency department visits, and inpatient stays (Figure S1). Per patient per month costs for 1L health care were primarily costs to Medicare ($8080), with patient liability costs of $1218, and other payer costs of $75 (data not shown). Similarly, Medicare, patient, and other payer PPPM costs for 2L and 3L care were, respectively, $7742, $1158, and $43, and $7655, $1121, and $27 (data not shown).

### 3.6 Survival

Of the 5303 patients who did not receive chemotherapy, 1.9% survived to the end of follow‐up, which was of a mean duration of 4.5 months. Of the 6509 patients who received 1L chemotherapy, the mean duration of follow‐up was 14.8 months, and 9.3% were alive at study completion.

Median survival from the 1L, 2L, and 3L index therapy dates was, respectively 9.2, 6.0, and 5.7 months (Table 3). Median survival was, 8.9, 7.8, and 6.5 months for carboplatin‐based regimens, and 10.8, 7.7, and 7.3 months for cisplatin regimens, respectively (Table 3). Median survival for any line of therapy with paclitaxel‐ and topotecan‐based regimens was in the range 3.4‐5.8 months. Median survival from the 1L index date for patients diagnosed at Stage I/II, III, and IV was 18.3, 11.6, and 8.0 months, respectively (data not shown). Corresponding values from the 2L index date were 11.8, 7.6, and 5.4 months, and from the 3L index date 10.2, 6.0, and 5.3 months (data not shown). Small cell lung cancer was the recorded cause of death in 88.7%. 92.1%, 93.5% of cases treated with 1L, 2L, and 3L therapies, respectively (data not shown).

**Table 3:** Survival from index therapy date, by line of therapy^a^

	1L		2L		3L	
	All (N = 6509)	Stage IV (N = 3986)	All (N = 2238)	Stage IV (N = 1429)	All (N = 679)	Stage IV (N = 419)
All therapies	9.2 (9.0‐9.4)	8.0 (7.8‐8.2)	6.0 (5.8‐6.4)	5.4 (5.0‐5.7)	5.7 (5.1‐6.0)	5.3 (4.7‐5.7)
Carboplatin‐based	8.9 (8.7‐9.1)	8.0 (7.7‐8.3)	7.8 (7.3‐8.4)	7.0 (6.4‐7.5)	6.5 (5.4‐7.7)	5.3 (4.4‐6.5)
Cisplatin‐based	10.8 (10.1‐11.4)	8.7 (8.1‐9.1)	7.7 (6.8‐8.7)	6.1 (5.2‐6.6)	8.3 (6.1‐11.6)	7.3 (5.4‐10.4)
Docetaxel‐based	10.1 (1.2‐19.0)	10.1 (1.2‐19.0)	4.0 (3.0‐4.9)	4.1 (2.9‐4.9)	3.4 (2.2‐6.8)	6.2 (2.7‐8.8)
Irinotecan‐based	5.1 (2.5‐9.2)	5.2 (1.8‐11.0)	4.9 (4.1‐6.1)	4.5 (3.4‐6.2)	5.2 (3.4‐6.0)	5.7 (3.1‐6.2)
Paclitaxel‐based	3.4 (2.4‐10.5)	4.4 (1.8‐10.5)	4.1 (3.6‐4.8)	4.1 (3.6‐4.7)	4.5 (4.0‐5.8)	4.5 (3.7‐6.4)
Topotecan‐based	5.8 (2.7‐8.3)	4.9 (1.7‐9.2)	4.6 (4.1‐4.9)	4.4 (3.8‐4.8)	5.0 (4.4‐5.7)	4.4 (3.7‐5.0)
Other	7.8 (7.0‐9.0)	6.7 (5.7‐7.4)	5.1 (4.2‐5.8)	4.6 (3.6‐5.6)	5.8 (4.5‐6.8)	5.5 (3.1‐6.8)

Abbreviations: 1L, first line; 2L, second line; 3L, third line.

a Values are presented as median (95% CI) months. Survival is shown from the index therapy date (1L, 2L, or 3L).

## 4 DISCUSSION

The SEER‐Medicare data presented here describe standard chemotherapy treatment of elderly patients with SCLC during 2007‐2014. Just over half (55%) received 1L chemotherapy, almost all platinum‐based, with 1L treatment typically lasting about 5‐6 months. The trend for 2L and 3L chemotherapy regimens was away from platinum‐based and towards a multiplicity of regimens. Each patient incurred total health care costs per month of about $9000 during 1L, 2L, and 3L treatment.

These results follow a series of previous SEER^17^, ^18^ and linked SEER‐Medicare^15^, ^19^, ^20^ database studies of SCLC patients, presenting data covering the period 1973‐2013.^15^, ^17^, ^18^, ^19^, ^20^ In the period 2007‐2014 in this study, 52% of the patients were female, compared with 42% in 1973‐2002, 48% in 1998‐2013, and 49% in 2000‐2008, illustrating the plateauing of the trend towards an increasing proportion of women with SCLC.^15^, ^17^, ^20^ As noted by Govindan et al, the proportion of women with SCLC increased from 28% in 1973 to 50% in 2002.^17^ Treatments have remained static during these time periods. In an analysis of SEER‐Medicare data for 2000‐2008, 86% of the patients newly diagnosed with extensive‐stage SCLC received 1L chemotherapy, most frequently carboplatin or cisplatin plus etoposide.^20^ Fewer than half (43%) of the patients who received 1L chemotherapy received a 2L regimen, and 18% received a 3L regimen—which compares with 36% and 11% of stage IV patients in this study.

Similar treatment patterns have been reported in other US observational studies.^21^, ^22^ In a study of an international database of medical chart reviews from 2014‐2016, 75% of US patients diagnosed with extensive‐stage SCLC received 1L therapy, 20% received 2L therapy, and 5% received 3L therapy.^22^ First‐line therapy was primarily (87%) a combination of a platinum and etoposide; topotecan was the most commonly used 2L chemotherapy. A study based on community electronic medical records from 2011‐2016 reported data for 334 patients who received 3L therapies.^21^ Almost all received 1L platinum‐based therapy, while for 2L therapy 49% received topotecan and 34% received platinum‐based therapy. For 3L treatment, platinum‐based regimens were used for 23% of patients.^21^

Rates of health care use and costs in this study are comparable to those reported in previous SEER studies. In SEER‐Medicare data of years 2000‐2008 for patients diagnosed with extensive‐stage SCLC, office visits were proportionately the largest cost driver, accounting for 43% of all disease‐related costs.^20^ In this study, outpatient costs were about 50%‐60% of total health care costs during chemotherapy treatment. In the 2000‐2008 study, the mean number of all‐cause office visits was 44.3^20^—compared with 48.4 outpatient visits during 1L treatment of stage IV patients in this study. In other SEER‐Medicare studies, average monthly costs of chemotherapy for a typical 72‐year‐old in the 6 months after diagnosis were in the range of about $7500‐$10 700 (in 2006 USD).^19^ Patient liability costs represented 14%‐16% of these costs. In the study of the SEER‐Medicare database for years 1998‐2013, total monthly costs of the initial phase of chemotherapy for a representative 70‐year‐old patient with extensive‐stage SCLC were $6713 (in 2017 USD).^15^

In SEER‐Medicare data for 2000‐2008, survival of patients from the date of diagnosis of extensive‐stage SCLC was a median of 7.4 months.^20^ In this study, median survival was 9.2 months from initiation of 1L treatment, and 6.0 months from initiation of 2L‐treatment. By comparison, in a systematic review of clinical trials published between 1984 and 2011, median survival following 2L therapy was 6.7 months.^9^ Standard chemotherapies have reached a plateau of effectiveness,^3^, ^23^ and new treatment strategies are needed to improve survival among SCLC patients. Clinical trials of immunotherapies have shown improved response rates in patients whose disease may no longer respond to cytotoxic therapy.^24^, ^25^, ^26^ Immunotherapy alone or in combination with 1L chemotherapy or in relapsed SCLC, may become a new option for the management of SCLC. Furthermore, patients on 3L treatment have high unmet medical needs given the short overall survival observed in this study. The recent approval of pembrolizumab for patients with metastatic SCLC with disease progression on or after platinum‐based chemotherapy and at least one other prior line of therapy could be a significant treatment breakthrough. Survival outcomes for patients with SCLC may improve in the coming years as a result of the developments in and introduction of new immunotherapies.

Since this was an analysis of a linked SEER‐Medicare data set, it was limited to patients ≥65 years. SEER data for patients diagnosed with limited stage SCLC in 1983‐1998 indicate that the median age at diagnosis was 67 years.^18^ In the study based on US community medical records, the mean age at SCLC diagnosis was 64 years.^21^ In the study of an international database of medical chart reviews, patients were diagnosed with extensive‐stage SCLC at an average age of 66 years (in addition, 34% were female, a much lower proportion than the approximately 50% in the United States).^22^ The costs in this study did not include prescription drug costs, ie, Medicare Part D prescription drug claims. Although available, the Part D prescription drug claims were not included in this analysis. During the study period approximately only half of the patients with Medicare Part A and B FFS coverage had Part D coverage. Furthermore, the majority of chemotherapy occurrences are covered and captured by Medicare Parts A and B (part A covers chemotherapy if the patient has cancer and is a hospital inpatient; part B covers chemotherapy if the patient is a hospital outpatient or a patient in a doctor's office or freestanding clinic). However, the lack of oral prescription drug data—including oral targeted agents (topotecan)—suggests that costs may have been underestimated. Average prescription drug costs for patients enrolled in Medicare Part D during 2007‐2013 were $1050 per month during the terminal phase of treatment of a typical patient of age 70.^15^

## 5 CONCLUSION

First‐line chemotherapy for SCLC was primarily platinum‐based, consistent with guidelines, at a health care cost of about $60 000. A plethora of different regimens was used for 2L and 3L chemotherapy, reflecting the lack of an accepted standard of care for these patients. Median survival from the start of chemotherapy was 9.2 months. The poor survival outcomes and high costs of care presented here are consistent with those reported in earlier studies. Median survival for patients receiving standard chemotherapy for metastatic SCLC has remained in the range of 8‐11 months for over 20 years.^3^, ^23^ These data provide a historical benchmark with which to assess the costs of the anticipated new immunotherapies and their effects on survival, as well as on the burden of SCLC on patients and on the health care system.

## Data Availability

The data that support the findings of this study are available in the SEER database, available at http://seer.cancer.gov.^16^
