# Supplementary material for: Chemotherapy treatments, costs of care, and survival for patients diagnosed with small cell lung cancer: A SEER‐Medicare study
Source: Cancer Med. 2019 Oct 31;8(18):7613–22. doi: 10.1002/cam4.2626 (PMC6912057; doi:10.1002/cam4.2626)
Supplement: Supplementary file 1 [file CAM4-8-7613-s001.docx]

Chemotherapy Treatments, Costs of Care, and Survival for Patients Diagnosed with Small Cell Lung Cancer: A SEER-Medicare Study

Changxia Shao^a^, Jinghua He^b^, Sumesh Kachroo^c^, and Fan Jin^d^

## Appendix

Table A.1 Patient demographic and clinical characteristics for stage IV patients ^A^

|  | Total  (N=7,797) | No chemotherapy  (N=3,811) | 1L  (N=3,986) | 2L  (N=1,429) | 3L  (N=419) |
| --- | --- | --- | --- | --- | --- |
| Age, mean years (SD) | 74.6 (6.2) | 75.6 (6.5) | 73.6 (5.7) | 72.7 (5.2) | 72.0 (5.0) |
| Female | 3,915 (50.2) | 1,925 (50.5) | 1,990 (49.9) | 710 (49.7) | 221 (52.7) |
| Race |  |  |  |  |  |
| White | 6,993 (89.7) | 3,373 (88.5) | 3,620 (90.8) | 1,279 (89.5) | 377 (90.0) |
| Black | 554 (7.1) | 307 (8.1) | 247 (6.2) | 96 (6.7) | 28 (6.7) |
| Asian | 212 (2.7) | 112 (2.9) | 100 (2.5) | * | * |
| Other/unknown | 27 (0.3) | 12 (0.3) | 15 (0.4) | * | * |
| CCI score, mean (SD) | 0.91 (1.34) | 1.01 (1.47) | 0.82 (1.20) | 0.75 (1.10) | 0.70 (1.13) |
| Region |  |  |  |  |  |
| Midwest | 1,170 (15.0) | 559 (14.7) | 611 (15.3) | 200 (14.0) | 67 (16.0) |
| Northeast | 1,376 (17.6) | 659 (17.3) | 717 (18.0) | 282 (19.7) | 83 (19.8) |
| South | 2,671 (34.3) | 1,272 (33.4) | 1,399 (35.1) | 467 (32.7) | 122 (29.1) |
| West | 2,580 (33.1) | 1,321 (34.7) | 1,259 (31.6) | 480 (33.6) | 147 (35.1) |
| Tumor size, mean size cm (SD) ^C^ | 5.47 (6.29) | 5.55 (6.09) | 5.39 (6.46) | 5.27 (5.51) | 5.69 (7.29) |

1L, first line; 2L, second line; 3L, third line; CCI, Charlson Comorbidity Index; SD, standard deviation

^A^ Values are presented as N (%) unless otherwise indicated.

^B^ Mean CCI score presented in 2014 macro.

^C^ Data available for 5,076 (65.1%) of all stage IV patients, 2,319 (60.9%) of patients receiving no chemotherapy, 2,757 (69.2%) of patients receiving 1L therapy, 1,009 (70.6%) of patients receiving 2L therapy, and 305 (72.8%) of patients receiving 3L therapy.

* Cell counts are suppressed according to CMS cell size suppression policy.

Table A.2 Duration of chemotherapy, by line of therapy ^A^

|  | 1L  (N=6,509) | 2L  (N=2,238) | 3L  (N=679) |
| --- | --- | --- | --- |
| Carboplatin-based | 5.9 (5.8, 5.9) | 4.8 (4.6, 5.0) | 5.4 (4.9, 6.1) |
| Carboplatin monotherapy | 5.1 (4.9, 5.4) | 4.1 (3.7, 4.5) | 3.5 (1.2, 5.4) |
| Carboplatin + etoposide | 5.9 (5.9, 5.9) | 4.7 (4.5, 5.0) | 5.4 (4.7, 6.6) |
| Carboplatin + irinotecan | - | - | - |
| Carboplatin + others | 5.6 (5.1, 6.1) | 5.1 (4.7, 5.5) | 5.8 (4.4, 6.5) |
| Cisplatin-based | 5.3 (5.2, 5.4) | 4.2 (4.0, 4.7) | 5.3 (4.5, 6.2) |
| Cisplatin monotherapy | 4.7 (3.8, 5.2) | 3.5 (3.0, 3.9) | 6.5 (3.0, 10.0) |
| Cisplatin + etoposide | 5.4 (5.2, 5.4) | 4.5 (4.0, 4.7) | 5.3 (2.3, 6.2) |
| Cisplatin + irinotecan | - | - | - |
| Cisplatin + others | 5.1 (4.0, 6.8) | 4.9 (4.0, 5.4) | 5.1 (4.5, 6.6) |
| Docetaxel-based | 8.2 (-) | 4.7 (3.5, 6.4) | 5.4 (3.0, 5.6) |
| Irinotecan-based | 4.9 (3.9, 7.0) | 5.6 (5.0, 6.2) | 4.8 (3.3, 5.5) |
| Paclitaxel-based | 4.4 (3.0, 7.9) | 5.4 (4.7, 5.8) | 5.0 (4.3, 6.2) |
| Topotecan-based | 5.6 (3.1, 8.6) | 4.6 (4.2, 5.0) | 4.2 (3.9, 4.8) |
| Other | 4.1 (3.7, 4.7) | 4.3 (3.8, 5.1) | 5.0 (4.0, 5.8) |

1L, first line; 2L, second line; 3L, third line

^A^ Values are presented as median months (95% CI).

Table A.3 Time to next chemotherapy, by line of therapy ^A^

|  | 1L  (N=6,509) | 2L  (N=2,238) | 3L  (N=679) |
| --- | --- | --- | --- |
| Carboplatin-based | 14.5 (13.3, 16.5) | 15.7 (13.2, 20.9) | 14.4 (7.7, -) |
| Carboplatin monotherapy | 12.1 (10.3, 15.2) | - (7.2, -) | 6.8 (1.2, -) |
| Carboplatin + etoposide | 15.6 (14.1, 17.2) | 15.6 (12.8, 25.5) | 23.6 (7.9, 23.6) |
| Carboplatin + irinotecan | - | - | - |
| Carboplatin + others | 11.2 (9.6, 12.7) | 15.3 (10.0, 19.1) | 10.1 (6.5, -) |
| Cisplatin-based | 18.1 (15.8, 21.7) | 11.7 (8.6, 16.5) | 7.5 (6.4, 10.1) |
| Cisplatin monotherapy | 18.2 (11.2, -) | 4.3 (3.3, -) |  |
| Cisplatin + etoposide | 18.9 (16.1, 23.6) | 13.4 (9.5, -) | 7.0 (2.3, 11.7) |
| Cisplatin + irinotecan | - | - | - |
| Cisplatin + others | 8.7 (7.0, -) | 8.6 (6.5, 15.2) | 7.4 (5.6, 9.2) |
| Docetaxel-based | - (-) | - (6.4, -) | 7.2 (3.1, -) |
| Irinotecan-based | 11.4 (3.9, -) | 10.0 (8.0, 29.9) | 12.9 (5.1, -) |
| Paclitaxel-based | 7.8 (4.4, -) | 9.8 (8.4, 13.4) | 9.1 (6.4, 11.2) |
| Topotecan-based | 15.9 (8.4, 15.9) | 9.2 (7.3, 10.5) | 7.0 (5.1, -) |
| Other | 10.8 (8.6, 21.4) | 12.2 (7.9, 14.9) | 6.8 (5.9, 9.6) |

1L, first line; 2L, second line; 3L, third line

^A^ Values are presented as median months (95% CI).

Figure A.1 Mean cost per patient, by setting and therapy type for (A) 1L, (B) 2L, and (C) 3L patients

Costs presented in 2016 USD.

1L, first line; 2L, second line; 3L, third line
